# Highlight report: Metabolomics in hepatotoxicity testing 

**DOI:** 10.17179/excli2017-1041

**Published:** 2017-12-21

**Authors:** Ahmed Ghallab

**Affiliations:** 1 Forensic Medicine and Toxicology Department, Faculty of Veterinary Medicine, South Valley University, Qena, Egypt

## ⁯⁯

Hepatotoxicity is one of the most frequent forms of systemic toxicity of drugs and a leading cause for drug withdrawal from the market. To improve the possibilities to predict hepatotoxicity, Tzutzuy Ramirez and colleagues from BASF in Ludwigshafen recently tested a metabolomics based *in vitro* system (Ramirez et al., 2017[20]). They exposed HepG2 cells to 35 test compounds and applied LC-MS/MS as well as GC-MS to quantify 89 metabolites in the culture medium supernatant and 194 intracellular metabolites. A main focus was to determine quality criteria, such as reproducibility and concentration dependency of the test system. The relative standard deviations of technical replicates were in the range of 5-10 %, while controls from different days were between 10 and 15 % (Ramirez et al., 2017[20]). This is an excellent reproducibility for an *in vitro* system. Moreover, convincing concentration-response relationships were obtained and metabolite patterns could be associated with specific mechanisms of toxicity, such as peroxisome proliferation and liver enzyme induction or inhibition (Ramirez et al., 2017[20]). Therefore, the HepG2 metabolomics technique represents a promising new candidate in the field of *in vitro* hepatotoxicity prediction. Limitations are that the HepG2 cells show major metabolic differences compared to human hepatocytes. Moreover, the present study did not yet include a systematic comparison of negative and positive controls at *in vivo *relevant concentrations, which remains a challenge for the future. 

Currently, mechanisms of hepatotoxicity represent a major focus in toxicological research (Kyriakides et al., 2016[18]; Ghallab, 2015[7]; Ramachandran et al., 2015[19]; Chen et al., 2015[4]; Campos et al., 2014[3]; Hammad et al., 2014[14]; Hassan, 2016[15]; Stöber, 2015[23]). Despite progress in the field of stem cell research (Gómez-Lechón and Tolosa, 2016[12]; Godoy et al., 2016[11]; Cameron et al., 2015[2]) and studies with cell lines (Tolosa et al., 2015[25]; Hewitt et al., 2007[16]; Godoy et al., 2013[10]), primary hepatocytes still remain a gold standard (Reif et al., 2015[22]; Grinberg et al., 2014[13]; Stöber, 2015[23]; Ghallab, 2015[6]; Arbo et al., 2016[1]). Moreover PBPK modeling (Ghallab, 2015[8]; Reif et al., 2017[21]; Thiel et al., 2015[24]) and spatio-temporal models (Ghallab et al., 2016[9]; Vartak et al., 2016[26]; Jansen et al., 2017[17]; Friebel et al., 2015[5]) have supported our understanding of the mechanisms of liver toxicity. The next year will show whether the novel HepG2 metabolomics assay can be integrated into useful test batteries for a better prediction of human hepatotoxicity. 
